# A Molecular Interaction Analysis Reveals the Possible Roles of Graphene Oxide in a Glucose Biosensor

**DOI:** 10.3390/bios9010018

**Published:** 2019-01-28

**Authors:** Tony Sumaryada, Muhammad Sandy Gunawan, Salahuddin Perdana, Sugianto Arjo, Akhiruddin Maddu

**Affiliations:** 1 Department of Physics, Bogor Agricultural University, Bogor 16680, Indonesia; sandyz.boyz@gmail.com (M.S.G.); salahuddinperdana@gmail.com (S.P.); akhiruddin@apps.ipb.ac.id (A.M.); 2 Program Studi Pendidikan Fisika, FKIP, Universitas HAMKA, Jakarta 13830, Indonesia; s.arjo@uhamka.ac.id

**Keywords:** graphene oxide, biosensor, glucose oxidase, molecular docking, mesoscopic system

## Abstract

In this paper, we report the molecular docking study of graphene oxide and glucose oxidase (GOx) enzyme for a potential glucose biosensing application. The large surface area and good electrical properties have made graphene oxide as one of the best candidates for an enzyme immobilizer and transducer in the biosensing system. Our molecular docking results revealed that graphene oxide plays a role as a GOx enzyme immobilizer in the glucose biosensor system since it can spontaneously bind with GOx at specific regions separated from the active sites of glucose and not interfering or blocking the glucose sensing by GOx in an enzyme-assisted biosensor system. The strongest binding affinity of GOx-graphene oxide interaction is −11.6 kCal/mol and dominated by hydrophobic interaction. Other modes of interactions with a lower binding affinity have shown the existence of some hydrogen bonds (H-bonds). A possibility of direct sensing (interaction) model of glucose by graphene oxide (non-enzymatic sensing mechanism) was also studied in this paper, and showed a possible direct glucose sensing by graphene oxide through the H-bond interaction, even though with a much lower binding affinity of −4.2 kCal/mol. It was also found that in a direct glucose sensing mechanism, the sensing interaction can take place anywhere on the graphene oxide surface with almost similar binding affinity.

## 1. Introduction

Graphene is a low dimensional mesoscopic system which is constructed from hexagonally arranged sp^2^ carbon atoms network [1,2,3,4]. This two-dimensional sheet of carbon allows direct interaction between carbon atoms and its environment, which potentially can be exploited in the sensing mechanisms, such as biosensor [5,6,7,8,9,10], vapor sensor [8,11], gas adsorption [12], and optical sensor [13]. The large span of potential application and its powerful physical properties have made research on graphene one of the hottest topics in material sciences nowadays. 

The large surface area combined with a very good electrical conductivity has made graphene-based biosensors powerful in terms of sensing accuracy and selectivity. The excellent biocompatibility and non-toxic properties also favor graphene for biosensor applications. Utilizing graphene in biosensing system requires incorporation of other materials to build a graphene composite or thin film electrodes. Some materials such as Nafion, polyaniline, and gold nanoparticles have been combined with graphene to immobilize the enzyme and to facilitate the electrochemical sensing mechanism. Some applications of graphene in biosensors include cholesterol detection [14,15], DNA detection [16,17,18,19] and glucose biosensors [20,21,22,23,24,25].

The use of graphene in glucose biosensing systems has been utilized intensively for more than a decade. There are two methods of glucose biosensing widely known, first the enzyme-assisted, and second, the non-enzymatic (direct) mechanism. For the enzyme-assisted method, researchers have focused on synthesizing the graphene nanocomposite system using various combinations of materials (polymer, metal, and nanoparticles) to immobilize the glucose oxidase enzyme and to facilitate the electrochemical process in the biosensors [26]. Fu et al. [27] used a graphite nanosheet–Nafion composite film to modify the electrode in a glucose biosensing system. A thin film of chitosan containing graphene and gold nanoparticle has also been used to immobilize the glucose oxidase (GOx) enzyme [28,29]. A hybrid bionanocomposite consisting of GOx/Pt nanoparticles/graphene-chitosan has been reported to show a good amperometric response toward glucose sensing [30,31]. For a direct sensing mechanism (non-enzymatic process), some progress has been reported, such as in [32,33,34,35,36,37]. 

The molecular docking method is a computational approach to analyze the receptor-ligand interaction by utilizing a rigorous physical concept combined with the optimization and statistical method. Through a docking method, we can have a good prediction on the likelihood of a particular molecule in making a complex with another molecule. There are two types of docking, rigid docking, and flexible docking. In this paper, we use a rigid docking where the target structure is rigid, while the ligand structure is flexible. The docking simulation of graphene oxide with glucose oxidase in this paper was inspired by the experimental work by Wang et al. [25], where the nitrogen-doped graphene was able to help the electron transfer from the FAD (flavin adenine dinucleotide) in the deeply seated cavity of the GOx enzyme to the electrode surface as shown by the well-defined redox peaks in the cyclic voltammogram. Such a nice experimental result would not be possible if the interaction of graphene and GOx occurred at the region close to the center redox of the enzyme (analyte active sites). The molecular interaction analysis here is aimed to evaluate the binding sites and affinities between graphene oxide and GOx enzyme to check whether or not the center redox cavity of the enzyme is blocked by graphene oxide. 

The aim of this research is to elucidate the molecular interaction between graphene oxide with GOx enzyme and glucose with graphene oxide to explore the graphene oxide’s potential in a biosensing system, whether as an enzyme immobilizer or as an active sensing material. There are some assumptions made at the beginning of this research. Firstly, we focus on the molecular interactions between graphene and GOx enzyme and not delving too much into the interaction between graphene and other materials, such as Nafion, polyaniline, gold nanoparticles, and etc. The blind docking method was chosen to enclose the whole surface of GOx enzyme and to identify the binding sites of graphene oxide as compared to the binding (active) sites of β-d-glucose. Secondly, we assume that the interaction between GOx with graphene oxide will not change the conformation of GOx in its native state and do not interfere with the glucose sensing process in the active sites of GOx. Although in reality the binding of graphene oxide with GOx enzyme in an aqueous condition most likely will change the enzyme’s conformation, but for the simplicity we assumed here that the adsorption of enzyme on graphene oxide surface is solely based on rigid docking calculation, without considering the dynamics of the complex in a particular environment (solvent). Further evaluations and more precise simulation of GOx-graphene oxide complex’s structural integrity through a molecular dynamics simulation [38] and molecular mechanics method are planned in the future. We also conducted a preliminary study of a possible direct biosensing mechanism or non-enzymatic process, where the glucose (β-d glucose) molecule directly interacts with graphene oxide. The binding affinities and binding sites of this mechanism are our interest.

## 2. Materials and Methods

### 2.1. Enzyme Preparation

**Figure 1:**
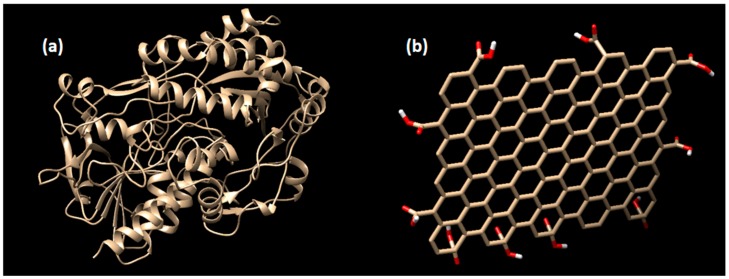
The structure of (**a**) glucose oxidase enzyme receptor (PDB: 1CF3), and (**b**) the graphene oxide. Note that the pictures are not to scale.

The structure of glucose oxidase for the enzyme receptor was downloaded from www.rcsb.org with PDB code of 1CF3 [39]. This enzyme structure was obtained from *Aspergillus Niger* bacteria. The enzyme structure was cleaned from water molecules, natural ligand, and other residual substrates using the Chimera program [40], then it was saved in *.pdb format. The docking preparations for the enzyme receptor were done using Autodock Tools ADT 1.5.6 [41]. In this software, the polar hydrogen atoms and Gasteiger charges were added before converted into the *.pdbqt format. The structure of the enzyme receptor and the graphene oxide are shown in Figure 1.

### 2.2. Ligand Preparation

The two-dimensional structure of graphene oxide (PubChem CID:124202900) was downloaded in the *.sdf format from https://pubchem.ncbi.nlm.nih.gov. The 2D structure then converted into a 3D structure using an online server at https://www.mn-am.com/online_demos/corina_demo. As we know, there are three kinds of graphene, pristine graphene, graphene oxide, and reduced graphene oxide [42]. The 3D structure of graphene oxide converted into the *.pdb format using Chimera. The polar hydrogen bonds and the Gasteiger charges were added to the ligand before it was saved in the *.pdbqt format by using Autodock tools ADT 1.56. 

### 2.3. Blind Docking Simulation

Since there is only limited information regarding the binding of graphene oxide on GOx, we had to perform a blind docking method to identify the possible binding modes. The blind docking was performed using Autodock Vina software [43] to find the best interaction mode with the strongest affinity or ΔG. The grid box size was set to x = 74 Å, y = 70 Å, and z = 54 Å, with the origin point set at x = 38.78, y = 6.43, and z = 53.088. All simulations were done on a desktop computer with Intel Core i7-3770, 3.40 GHz, and 12 GB of RAM, under Ubuntu 14.04 LTS 64 bit platform.

### 2.4. Biosensing Mechanism and Model

The sensing mechanism of glucose by GOx can be expressed as

β-d-glucose + O_2_ → gluconic acid + H_2_O_2_ → 2H^+^ + O_2_ + 2e^−^. (1)

**Figure 2:**
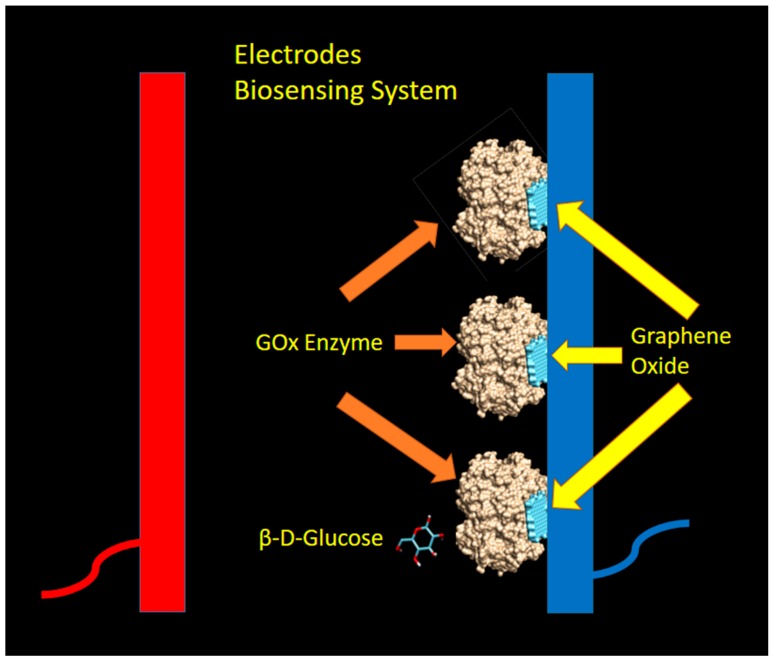
The model of biosensing mechanism utilizing glucose oxidase (GOx) enzyme and graphene as Immobilizer. The light blue color denotes the graphene oxides, while the gold color represents the GOx enzyme. The dark blue and red color bars represent the electrodes (**dark blue** for the cathode, and **red** color for anode).

The glucose oxidase enzyme catalyzes the oxidation of β-d-glucose into d-glucono-1,5-lactone, which is then hydrolyzed to gluconic acid and hydrogen peroxide (H_2_O_2_). The hydrogen peroxide then dissociates into 2H^+^, O_2_, and two free electrons, which can be captured by the electrode and measured by the electronic instrument [44]. The sensing model (and assumptions) used in this paper are shown in Figure 2 below.

In this proposed biosensing system, the graphene layers were attached to the cathode (by coating technique) and expected to bind with GOx enzymes with some kind of non-covalent interaction at particular binding sites. By assuming the GOx-graphene oxide interaction occurred at a different pocket as compared to the active sites of β-d-glucose, the sensing mechanism as explained by Equation (1) was not disturbed and still took place. In this mechanism, graphene oxide was acting as an enzyme immobilizer which facilitates, and not interferes with the glucose sensing activity by GOx enzyme.

## 3. Results and Discussion

### 3.1. Molecular Interaction Analysis of Graphene Oxide and GOx

**Figure 3:**
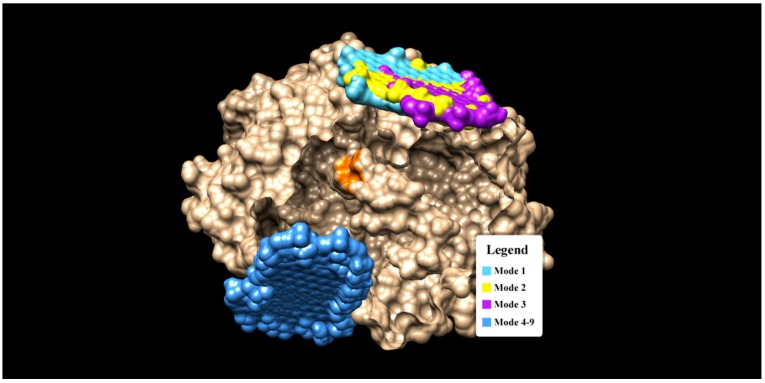
The binding modes of Graphene oxide on a GOx enzyme (gold color). Note that the orange spot (region) in the center of GOx indicates the active sites (interaction pocket) of β-d glucose.

The catalytic sites of GOx enzyme which interacts with β-d glucose are GLU 412, HIS 516, HIS 559 [45]. Through a blind docking simulation, we are interested in identifying the binding sites of graphene on GOx. If graphene interacts with those catalytic sites then, it will act as a competitor for β-d glucose and negates the possibility of using graphene in biosensing applications. On the other hand, if graphene does not interact with the active sites of glucose, then graphene acts as a non-competitive ligand to glucose and can be used as a GOx enzyme immobilizer in an enzyme-assisted biosensor, such as in [26]. The Autodock Vina results are given in Table 1, while the interaction modes are shown in Figure 3.

**Figure 4:**
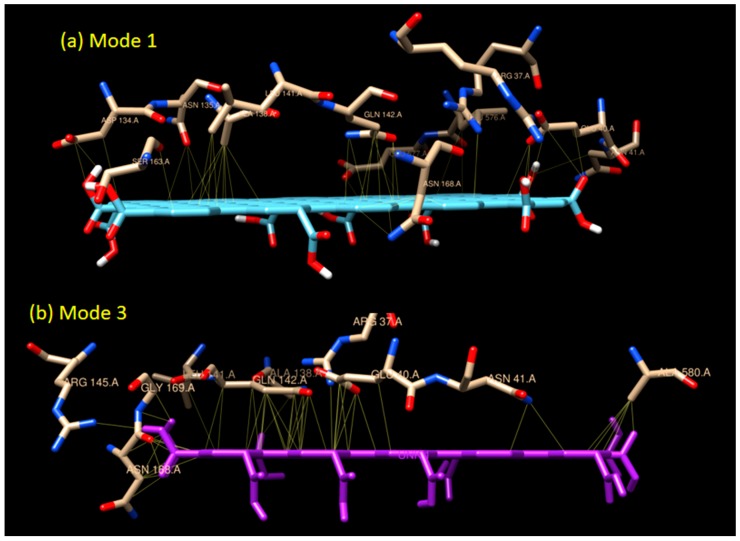
The interaction models of graphene oxide and GOx enzyme in (**a**) Mode 1 and (**b**) Mode 3. The detailed list of amino acids involved can be seen in Table 1.

It was found that mode 1 to 3 have almost similar binding sites (see Figure 3), while mode 4 to 9 cluster themselves in the completely different binding sites. Notice that none of the graphene oxide’s binding sites (modes) overlapped with the binding site of glucose (orange spot in Figure 3). There are two interaction modes (mode 1 and 3) which show good prospects as an enzyme immobilizers. Mode 1 has the strongest binding affinity of −11.6 kCal/mol, but there are no H-bonds present in this mode. The binding interaction in mode 1 is dominated by the hydrophobic interaction (a weak non-covalent interaction) and involves 19 residues (see Figure 4). The important role of hydrophobic interaction in making a stable complex in mode 1 is in accordance with [46], where the hydrophobic interactions favor a more productive interaction between the two redox molecules (biomolecules).

**Figure 5:**
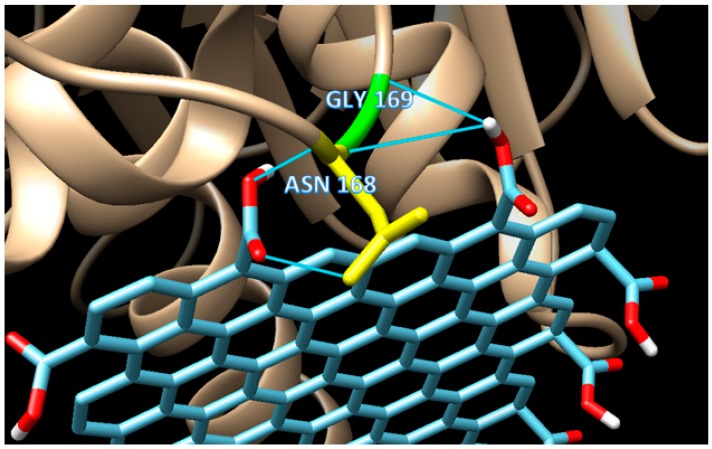
The detailed interactions of mode 3, which shows the occurrence of double H-bonds, one from GLY169 (H-bond distance of 3.149 Å), and one from ASN168 (H-bond distance of 2.859 Å).

Unlike mode 1, the binding affinity of mode 3 is slightly weaker (−10.5 kCal/mol). There are 18 residues involved in the hydrophobic interaction in addition to two residues involved in H-bond interaction. Two H-bonds (double bonds) present in mode 3, with one H-bond, occurred in GLY 169 (H-bond distance of 3.149 Å), and one in ASN 168 (H-bond distance of 2.859 Å) as seen in Figure 5. Based on their distances, those H-bonds are considered as a medium to weak H-bond since the distances are above 2.50 Å. The binding mode 3 is an example of a mixed-type of weak non-covalent (hydrophobic) and strong non-covalent (H-bond) interaction, which serves the possibility of using graphene oxide as the enzyme immobilizer for the biosensing system.

### 3.2. Direct Sensing of Glucose by Graphene Oxide: A Non-Enzymatic Mechanism

The large surface area has made graphene oxide very promising for biosensor applications due to its superior electronic and electrochemical properties [47]. Some research on non-enzymatic sensing of graphene have been done by [32,33,34,35,48,49]. It is interesting to check the possibility of direct glucose (β-d glucose) sensing by the graphene oxide through a simulation approach. We performed a blind-docking method to explore any possible interaction between the glucose molecule with the surface of the graphene oxide layer. The docking simulation was set by the grid box size of x = 30 Å, y = 24 Å, and z = 26 Å, and the origin point at x = 0.135, y = 0.041, and z = 0.018. The number of modes and exhaustiveness were set to 20 and 16 respectively. The docking results of this non-enzymatic sensing model are shown in Table 2.

**Figure 6:**
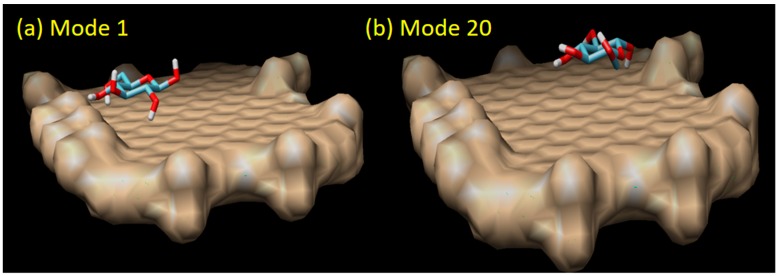
The direct sensing model of graphene oxide and glucose molecule in mode 1 (the strongest binding affinity of −4.20 kCal/mol) and mode 20 (the weakest binding affinity of −3.70 kCal/mol).

**Figure 7:**
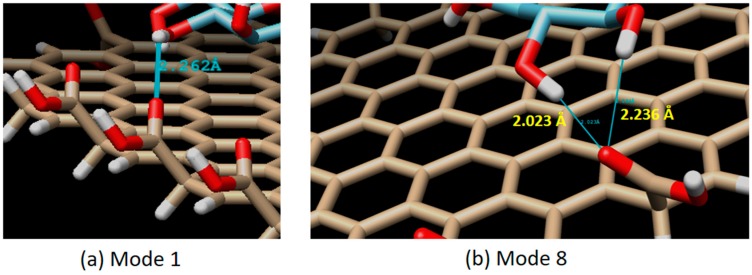
Interaction mode which has an H-bond. Single H-bond in mode 1 (H-bond distance of 2.262 Å), and a double H-bond in mode 8 (H-bond distance of 2.023 and 2.236 Å).

There are 20 binding modes identified between β-d-glucose and graphene oxide. The strongest interaction (mode 1) has the binding affinity of ΔG = −4.2 kCal/mol, while the weakest (mode 20) has ΔG = −3.7 kCal/mol (see Figure 6). The relatively small difference (about 13.5% of difference) between the strongest and the weakest ΔG value, and also the relatively small difference of the root mean square deviation (RMSD) between mode 1 and the rest indicate that the binding of β-d-glucose on graphene oxide surface can take place everywhere with relatively the same strength or sensitivity (around −4.0 kCal/mol). From 20 binding modes, only mode 1 and 8 which have H-bond interactions. In mode 1, one H-bond is identified with a distance of 2.262 Å. Mode 8 has two H-bonds, each with a distance of 2.023 and 2.236 Å (see Figure 7). Based on our result, it was found that the H-bonds in the direct glucose-graphene interaction are stronger (2.262 and 2.263 Å) as compared to the H-bonds present in GOx-graphene interaction (2.859 and 3.149 Å). This finding emphasizes that even though the enzyme-assisted mechanism produces a weaker H-bond interaction but overall it produces a stronger binding affinity (due to a large number of hydrophobic interaction), about three times bigger than the non-enzymatic mechanism.

## 4. Conclusions

In this work, we have performed a molecular interaction analysis based on the molecular docking results to explore the potential of graphene oxide in glucose biosensing applications. Two mechanisms were simulated in this paper, first the enzyme-assisted mechanism and second the non-enzymatic (direct) mechanism. Based on the simplified docking parameter assumptions used here, which in some cases might be favorable compared to experimentally achievable results, molecular docking analysis has revealed that graphene oxide can be used as an enzyme immobilizer as shown by its strong binding affinity (ΔG value) and its binding sites which do not overlap with the active sites of β-d-glucose on GOx enzyme. The binding of GOx enzyme with graphene oxide is mostly dominated by a weak non-covalent (hydrophobic) interaction with a small portion of a strong non-covalent (H-bond) interaction presents. Since graphene oxide binds in the different pocket than β-d-glucose, the binding of GOx and graphene oxide will not interfere the glucose sensing process in the active sites of GOx (GLU 412, HIS 516, HIS 559). At this stage of research, we only consider the binding between graphene oxide and GOx enzyme. The point mutation effect on the particular amino acid of GOx enzyme will definitely change the enzyme adsorption at the graphene surface, since the adsorption free energy of each amino acid is different. A molecular dynamics simulation will be conducted in the future to reveal the conformational ensemble of the adsorbed enzyme as shown by [38]. The molecular docking analysis of a direct interaction between β-d-glucose and graphene oxide shows a promising application of graphene oxide layer as an active biosensor material through a non-enzymatic mechanism. The relatively the same binding affinity of β-d-glucose on graphene oxide (around −4.0 kCal/mol), regardless of the position of interaction on the graphene oxide surface, offers an almost uniform glucose-probing sensitivity of the graphene oxide layer. Despite a much weaker binding affinity of β-d-glucose with graphene oxide as compared to the GOx-graphene oxide binding, the large surface area of graphene oxide with relatively the same sensitivity makes graphene oxide also promising to be developed into a non-enzymatic biosensor. The direct glucose sensing simulation here is only a preliminary study of a possible direct biosensing mechanism, where glucose (β-d glucose) molecules directly interact with graphene oxide. Further study must be done on this subject in the future. Unlike the drug discovery research where a high throughput virtual screening with multiple docking is common, the multiple docking of various analytes with graphene (or graphene oxide) to study the graphene’s selectivity, to the best of authors knowledge, has never been found in the literature and would be interesting to do in future.

## Figures and Tables

**Table 1:** The docking results of GOx-Graphene.

Mode	ΔG (kCal/mol)	Residue Involved in H-Bond	H-Bond Distance	Residue Involved in Hydrophobic Interaction
1	−11.6	-	-	ARG 37, GLU 40, ASN 41, ASP 134, ASN 135, ALA 138, TYR 139, LEU 141, GLN 142, ARG 145, SER 163, GLY 166, VAL 167, ASN 168, GLY 169, ARG 239, ASP 573, LEU 576, GLU 577
2	−11.0	-	-	ARG 37, GLU 40, ASN 41, PRO 42, ASP 134, ASN 135, ALA 138, LEU 141, GLN 142, ALA 162, SER 163, CYS 164, HIS 165, GLY 166, VAL 167, ASN 168, GLY 169, ARG 239, ASP 573, LEU 576, GLU 577, TYR 579, ALA 580
3	−10.5	GLY 169 ASN 168	3.149 2.859	ARG 37, GLU 40, ASN 41, PRO 42, ALA 138, TYR 139, LEU 141, GLN 142, ALA 143, GLU 144, ARG 145, GLY 166, VAL 167, THR 170, ASP 573, GLU 577, ALA 580, SER 581
4	−10.1	GLU 374 GLU 378	2.770 3.143	MET 305, SER 307, ILE 308, ASP 319, LEU 320, PRO 321, LEU 324, VAL 381, ALA 382, GLY 384, PHE 386, HIS 387, ASN 388, THR 389, THR 390, LYS 526, GLU 527
5	−9.80	ALA 382 ALA 382	2.832 2.782	MET 305, SER 307, ASP 319, LEU 320, PRO 321, LEU 324, GLU 378, VAL 381, ARG 383, GLY 384, PHE 386, HIS 387, ASN 388, THR 389, THR 390, LYS 526, GLU 527
6	−9.70	-	-	MET 305, LYS 306, SER 307, ASP 319, GLU 374, GLU 378, VAL 381, ALA 382, HIS 387, ASN 388, THR 389, THR 390, LYS 526, GLU 527
7	−9.50	-		MET 305, LYS 306, SER 307, ILE 308, ASP 319, LEU 320, GLU 374, GLU 378, VAL 381, ALA 382, ARG 383, HIS 387, ASN 388, THR 389, THR 390, LYS 526, GLU 527
8	−9.40	-	-	MET 305, SER 307, ASP 319, GLU 378, VAL 381, ALA 382, HIS 387, ASN 388, THR 389, THR 390, LYS 526, GLU 527
9	−9.30	SER 307 ASP 319	2.959 2.703	MET 305, LYS 306, ILE 308, LEU 320, GLU 378, VAL 381, ALA 382, HIS 387, ASN 388, THR 389, THR 390, LYS 526

**Table 2:** Docking results of graphene oxide and β-d-glucose in a direct sensing model.

Mode	ΔG (kCal/mol)	RMSD l.b (Å) *	RMSD u.b (Å) *
1	−4.20	0.000	0.000
2	−4.10	1.234	3.649
3	−4.10	1.059	4.093
4	−4.00	2.818	5.098
5	−4.00	1.272	2.009
6	−4.00	2.362	3.661
7	−4.00	1.254	2.314
8	−3.90	2.211	4.563
9	−3.90	1.502	3.750
10	−3.90	1.535	2.754
11	−3.90	3.528	5.889
12	−3.80	2.321	4.232
13	−3.80	2.426	4.128
14	−3.80	3.587	4.876
15	−3.80	2.106	4.095
16	−3.80	1.113	3.851
17	−3.80	2.604	3.853
18	−3.80	1.680	2.305
19	−3.80	1.789	3.733
20	−3.70	5.241	7.421

* Note: l.b stands for the lower bound, while u.b stands for the upper bound.
